# Low awareness of community-dwelling older adults on the importance of dietary protein: new insights from four qualitative studies

**DOI:** 10.1017/jns.2021.92

**Published:** 2021-12-07

**Authors:** Joost O. Linschooten, Marije H. Verwijs, Janne Beelen, Marian A. E. de van der Schueren, Annet J. C. Roodenburg

**Affiliations:** 1 Department of Food Science & Technology, HAS University of Applied Sciences, 's-Hertogenbosch, The Netherlands; 2 Department of Nutrition and Health, HAN University of Applied Sciences, Faculty of Health and Social Studies, Nijmegen, the Netherlands; 3 Sensory and Consumer Science, CSIRO Health and Biosecurity, North Ryde, NSW 2113, Australia

**Keywords:** Attitude, Community-dwelling older adults, Protein, Qualitative studies, Undernutrition

## Abstract

Meeting the recommended daily protein intake can be a challenge for community-dwelling older adults (CDOA). In order to understand why, we studied attitudes towards protein-rich products and healthy eating in general; identified needs and preferences, barriers and promotors and knowledge regarding dietary behaviour and implementation of high protein products. Attitudes towards protein-rich products and healthy eating were evaluated in focus groups (study 1, *n* 17). To gain insights in the needs and preferences of older adults with regard to meals and meal products (study 2, *n* 30), visual information on eating behaviour was assessed using photovoicing and verified in post-photovoice interviews. In studies 3 and 4, semi-structured interviews were conducted to identify protein consumption-related barriers, opportunities (*n* 20) and knowledge and communication channels (*n* 40), respectively. Risk of low protein intake was assessed using ProteinScreener55+ (Pro55+) in studies 2–4 (*n* 90). Focus groups showed that participants were unaware of potential inadequate dietary protein. Photovoicing showed that sixteen of thirty participants mainly consumed traditional Dutch products. In post-photovoice interviews, participants indicated that they were satisfied with their current eating behaviour. Barriers for adequate use of protein-rich products were ‘lack of knowledge’, ‘resistance to change habits’ and ‘no urge to receive dietary advice’. Promotors were ‘trust in professionals’ and ‘product offers’. Sixty-two percent had a low risk of low protein intake. CDOA feel low urgency to increase protein intake, possibly linked to low knowledge levels. A challenge for professionals would be to motivate older adults to change their eating pattern, to optimise protein intake.

## Introduction

During the past centuries, life-expectancy of humans increased significantly. Consequently, reaching an age of 90 years or more is becoming very common^(1,2)^. Looking at the Netherlands, the number of community-dwelling older adults (CDOA, aged 65 years or older) is expected to increase from almost 19 % in 2019 to 26 % in 2040^(3)^. In general, older adults have a higher risk of protein–energy malnutrition^(4)^, which could lead to loss of skeletal muscle mass, quality and strength (sarcopenia)^(5)^ and eventually to dependency in activities of daily living and decreased mobility^(6)^. Consequently, older adults are at higher risk to move into residential aged care^(7)^, subsequently resulting in higher healthcare costs^(8)^. Therefore, the Dutch government has developed several programmes stimulating older adults to live at home as long as possible^(9,10)^. Yet, this requires that older adults are vital and independent.

In order to remain vital at an older age, expert groups suggest a protein intake of at least 1⋅0 g protein/kg body weight per day (g/kg bw/d) for older adults, or even 1⋅2 g/kg bw/d when suffering from any acute or chronic disease^(11)^. Previous studies showed that a protein intake of >1⋅0 g/kg bw/d was associated with a protective effect against weight loss^(12)^ and with a lower risk of development for mobility limitations^(13,14)^ among CDOA. These and other studies suggest that the current recommended dietary allowance (RDA) (0⋅8 g/kg bw/d) is indeed too low to maintain optimal physical functioning in older adults^(4)^. Data from the Dutch National Food Consumption Survey (DNFCS)-Older adults 2010–2012 showed that 15⋅4 % did not meet the recommendation of 0⋅8 g/kg bw/d and approximately 50 % had an intake lower than 1⋅0 g/kg bw/d^(15)^. In this population, almost 70 % suffered from one or more chronic diseases, potentially causing even a higher risk of malnutrition considering the advice of 1⋅2 g/kg bw/d for this subgroup.

Analysis of protein intake per meal moment among Dutch community-dwelling older adults showed that protein intake reaches adequate levels at dinner, where breakfast and lunch are open for improving protein intake^(16)^. Furthermore, analysis of Dutch cross-sectional data^(17)^ illustrates that older adults (aged ≥70 years) with a low protein intake (<0⋅8 g/kg bw/d; ~15 %) consumed less protein in general and less animal protein at all eating occasions. Important protein sources in this population were dairy, meat and cereals. No specific determinants for low protein intake could be identified, but general characteristics of older adults with low protein intake were: following a diet, being obese and, to some extent, a lower frequency of drinking alcohol^(15)^. Further identification of the target population and characterisation of their daily habits is required to address this issue of optimising protein intake.

A widely used treatment to improve nutritional intake is prescription of oral nutritional supplements (ONS)^(18–20)^. Although these studies were effective in increasing protein intake in a clinical setting, most of these studies used ONS (or similar high protein products) in addition to a current daily dietary pattern. It can be questioned how older adults comply to the use of these products when interventional studies (and subsequently support) have ended^(19)^, as compliance mainly relies on patients’ will, information and support^(21)^. Furthermore, these products are often only available upon prescription by a dietitian and not in all countries covered by healthcare insurance. Finally, many of these products are not available in retail, which hinders incorporation of the use of ONS in the daily lifestyle for older adults living at home. Decreased compliance can also be linked to disliking ONS after repeated consumption^(22,23)^.

Apart from the population that depends on ONS use, for some older adults the ONS-dependent phase can potentially be postponed by an improved dietary pattern enriched in protein. The past few years promising data became available on the effectiveness of shifting towards foods higher in protein in CDOA who are doing their own groceries and preparing their own meals^(24)^. The use of protein-rich foods that are familiar to older adults might be an effective way to increase protein intake^(25,26)^ since familiarity with the type of food or the typical moment of consumption might enable the use of these products in a regular daily pattern. However, research shows that there is a lack of knowledge and awareness among older adults on the importance of an adequate protein intake is still low^(27,28)^.

### Aim of this study

The present study describes four studies with slightly different aims: the first study aimed to gain insights into CDOA's opinion towards healthy eating and their attitudes towards high protein products; the second study aimed to identify needs and preferences with regard to products, meals and meal products; the third studied the main barriers and promotors with regard to the use of high protein products and the fourth study investigated opportunities for modifications of current dietary behaviour and how this relates to the estimated protein intake. The present study aimed to combine the results of these four studies to provide more comprehensive insights into attitudes, barriers and behaviour of CDOA regarding protein consumption.

## Methods and material

### Four qualitative studies

To gain insights into opinions on healthy eating and attitudes towards high protein products, a qualitative research design was used for the collection and analysis of the data from participants from the ConsuMEER study (study 1)^(29)^. Subsequently, in three additional studies, qualitative research techniques were used. To identify needs and preferences with regard to products, meals and meal products, visual information on eating behaviour was collected using photovoicing (study 2). In studies 3 and 4, semi-structured interviews were conducted to identify protein consumption-related barriers, opportunities (*n* 20) and knowledge and communication channels (*n* 40), respectively. New participants were recruited for each subsequent study to reduce participant burden.

The following demographic data were collected for all studies: gender, age, marital status, body mass index (BMI), healthcare allowance (as an indicator for the level of income), the number of children and grandchildren and educational level.

### Participants

Study 1: the ConsuMEER study was a single-blind randomised controlled trial with 100 CDOA switching from self-prepared to commercially available home-delivered hot meals and dairy products during 28 d^(29)^. The intervention group (I) received meals and dairy products high in protein (≥20 energy%); the control group (C) received standard meals and dairy products low(er) in protein (<20 energy%). All products were provided free of charge (worth about 250 euro) and were chosen by participants themselves and delivered at home. Dietary intake was measured at baseline, 2 weeks (T1) and 4 weeks (T2) using a 3-d food diary. After study completion, all participants were invited to participate in a focus group to get a better understanding of their opinions regarding healthy eating and attitudes towards high protein products. A total of seventeen older adults participated across three focus groups. Detailed information on the study design of the ConsuMEER study can be found elsewhere^(29)^.

Studies 2, 3 and 4: for the three other studies, additional participants (*n* 90) were recruited through daily activity centres in the 's-Hertogenbosch region (The Netherlands) and through snowball sampling. None of the participants knew the researchers prior to participation. Inclusion criteria for these three studies were: aged 65 years and older, living at home, preparing at least four hot meals per week. In order to study possible differences between single and married older adults, an equal number of single and married subjects (one per household) were included in study 3 and study 4.

### ProteinScreener55+ (Pro55+)

In studies 2, 3 and 4, protein intake was assessed using the Pro55+^(30)^, an online tool to calculate the risk of a low protein intake (<1⋅0 g/kg bw/d). The outcome is based on ten questions on the consumption frequency and quantity of protein-rich products and is expressed as the relative chance on low protein intake (see Supplementary material). Subjects who score >30 % are considered to be at high risk for a low protein intake.

### Procedure and data collection

#### Focus group interviews (study 1)

A discussion guide was developed to explore the topics healthy eating and attitude towards high protein products. Three focus group sessions (*n* 17) were conducted in June 2017 at both HAS and HAN universities of applied sciences and were led by an independent researcher, supported by a research assistant taking minutes. All discussions (max 2 h) were audiorecorded. Prior to the start of the focus group interviews, participants had not been informed that the main aim of the ConsuMEER study was on the increase of protein consumption. During the discussion on attitudes towards high protein products, participants were presented a range of product packages with various health claims (e.g. related to fibre content, protein content, calcium content and plant sterols) to identify how familiar participants were with these claims. All findings were transcribed verbatim, subsequently coded, analyzed and summarised in a report by two independent researchers, in line with the focus group discussion guide.

#### Photovoicing (study 2)

To gain more insights into the needs and preferences of older adults with regard to food products and meals, photovoicing was applied to collect visual information on habitual dietary behaviour^(31)^. Food records and dietary history are considered to be very common and valuable dietary assessment methods, also in an older adult population^(32)^. However, photovoicing may provide additional data on consumption habits. A total of thirty participants was asked to take photos of everything they consumed for a period of three consecutive days (two weekdays and one weekend day). Photos were sent to the researchers via email, followed by a post-photovoice interview referring to participant's own photos to verify if they had captured total consumption and whether participants modified their consumption behaviour as a result of photographing their meals. Additional questions on consumption needs and preferences were asked (list available in Supplementary material). All data was collected between April and June 2019.

#### Semi-structured interviews (studies 3 and 4)

Semi-structured, face-to-face qualitative interviews were performed to gain deeper insights into barriers and promotors for the use of high protein products (study 3, *n* 20), and individual knowledge on protein to identify possibilities for the development of future communication strategies (study 4, *n* 40).

Two discussion guides were developed for the semi-structured interviews, one focusing on barriers and promotors of high protein products (study 3) and one focusing on the identification of knowledge gaps and communication channels (study 4), see Table 1. The interviews were conducted between April and July 2019 at the participants’ own homes. All interviews were audiorecorded, anonymized and transcribed by the interviewers^(33)^. For study 3, researchers prepared verbatim transcripts and coding trees for further analysis, and for study 4, researchers verified the summary of findings with the interviewee. In both studies, two interviewers listened to the recorded interviews separately and a formal coding process was performed using the topic guide as shown in Table 1. Both researchers manually assigned open codes to pieces of text with the same meaning, and subsequently codes were discussed to manage discrepancies by consensus. If necessary, new codes were adapted to coding trees and previous transcripts were recoded. Codes were grouped into themes and specified into categories. In case topics occurred that could not be grouped under the topics that were identified initially, extra categories were added in order to include possible niches in the analysis.

**Table 1.** Topics of the semi-structured interviews on barriers and promotors of high protein products (study 3) and on knowledge on dietary protein and communication channels (study 4)

Study 3	Study 4
Grocery shopping	*Main topic*
Meal preparation	Dietary protein and communication
Consumption pattern	
Healthy ageing	*Sub-topics*
Knowledge/influence	Knowledge on healthy nutrition
Communication channels	Knowledge on dietary protein
	Communication preferences

### Statistical analyses

Statistical analyses of data from the ConsuMEER study is described elsewhere^(29)^. Descriptive statistics were used to summarise participants’ characteristics and data were reported as means and standard deviations for continuous data and frequencies and percentages for categorical data. A logistic regression analysis was performed to test differences in the dichotomous variable (Pro55+ score ≤30 % *v*. > 30 %). All analyses were performed using SPSS 24 (IBM, Chicago, IL) and a *P*-value <0⋅05 was considered significant.

### Ethics

For study 1, ethical approval was obtained from the Ethical Advisory Board from the Radboud University Nijmegen Medical Centre, Nijmegen, The Netherlands [as part of the ConsuMEER study]. For studies 2–4, ethical and legal advice was obtained from the Ethical Advisory Committee from HAS University of Applied Sciences, 's-Hertogenbosch, The Netherlands. This committee had no official status at that time. Therefore, the advice was verified by the Ethical Advisory Board from the HAN University of Applied Sciences, Nijmegen, The Netherlands. It was judged not to fall within the remit of the Medical Research Involving Human Subjects Act (WMO) and ethical clearance was provided by the review board. Participation was voluntary and all participants provided written informed consent prior to participation. Participants were free to withdraw from the study at any time. All participants were informed to consult their general practitioner and/or a dietician in case of a high chance of a low protein intake and received a flyer from the Dutch Malnutrition Steering Group with additional information. All data was anonymized and stored on a protected server only accessible by selected members of the research team. Anonymized data can be made available upon request from the corresponding author. Due to privacy and ethical restrictions, data are not publicly available.

## Results

### Baseline characteristics

Out of 100 participants, 88 completed the ConsuMEER trial and were asked to voluntarily participate in one of three additional focus group interviews. A random selection of seventeen CDOA (twelve females/five males) participated in a focus group session, and they were considered to reflect the total study population from the ConsuMEER study (mean age 80⋅4 ± 6⋅8, mean BMI 27⋅9 ± 5⋅0 kg/m^2^). A detailed information of the ConsuMEER participants are described elsewhere^(29)^. Baseline characteristics of participants from studies 2, 3 and 4 are shown in Table 2; the overall mean age was 75⋅6 ± 7⋅8 years and mean BMI was 26⋅6 ± 3⋅5 kg/m^2^.

**Table 2.** Baseline characteristics of participants included in studies 2, 3 and 4 (frequencies and percentages; mean values and standard deviations)

	Study 2 (*n* 30)		Study 3 (*n* 20)		Study 4 (*n* 40)	
	*n*	%	*n*	%	*n*	%
Gender						
Male	12	40⋅0	6	30⋅0	17	42⋅5
Female	18	60⋅0	14	70⋅0	23	57⋅5
Age (years)						
Mean	72⋅0 (sd: 6⋅1)		75⋅3 (sd: 7⋅8)		78⋅4 (sd: 8⋅0)	
BMI (kg/m²)						
Mean	25⋅8 (sd: 2⋅1)		27⋅4 (sd: 4⋅9)		26⋅8 (sd: 3⋅4)	
Marital status						
Single	12	40⋅0	10	50⋅0	20	50⋅0
Married	18	60⋅0	10	50⋅0	20	50⋅0
Income^a^						
Low	9	30⋅0	9	45⋅0	22	55⋅0
High	21	70⋅0	11	55⋅0	18	45⋅0
Children						
Yes	26	86⋅7	18	90⋅0	37	92⋅5
No	4	13⋅3	2	10⋅0	3	7⋅5
Grandchildren						
Yes	21	70⋅0	17	85⋅0	36	90⋅0
No	9	30⋅0	3	15⋅0	4	10⋅0
Educational level^b^						
Low	11	37⋅0	9	45⋅0	20	50⋅0
Middle	6	20⋅0	9	45⋅0	16	40⋅0
High	13	43⋅0	2	10⋅0	4	10⋅0
ProteinScreener55+^c^						
Mean %	20⋅4 (sd: 25⋅4)		23⋅9 (sd: 29⋅9)		35⋅9 (sd: 29⋅4)	
Participants with a score >30 %	6	20⋅0	7	35⋅0	21	52⋅5

a Low income was defined as annual income <€28 500 for singles or <€35 000 for couples.

b Educational level was defined as ‘Low’ when participants had only completed primary education, lower vocational education and/or advanced elementary education; ‘Middle’ for completing intermediate vocational education, higher secondary education; and ‘High’ for higher vocational education and university.

c Subjects with Pro55+ score >30 % are considered to be at high risk for a low protein intake.

### ProteinScreener55+

All participants from studies 2, 3 and 4 were screened for the risk of low protein intake (defined as <1⋅0 g/kg bw/d) using the Pro55+ (Table 3). We observed a substantial difference in the risk of low protein intake between subjects with a low income compared with subjects with a higher income. No significant or non-significant but substantial differences could be identified. Since BMI is integrated in the Pro55+, this item cannot be considered as a relevant significant difference.

**Table 3.** Evaluation of pooled Pro55+ scores from participants from studies 2–4 (*n* 90) per baseline characteristics

	≤30 %, low risk^a^	>30 %, high risk^a^	*P*-value
	*n* 56	*n* 34	
Age	72⋅5 (67⋅3−80⋅8)	77⋅5 (71⋅0−83⋅3)	0⋅113
Gender			0⋅586
Male	23 (41⋅1 %)	12 (35⋅3 %)	
Female	33 (58⋅9 %)	22 (64⋅7 %)	
Marital status			0⋅954
Single	26 (46⋅4 %)	16 (47⋅1 %)	
Married	30 (53⋅6 %)	18 (52⋅9 %)	
BMI	26⋅0 (sd: 3⋅0)	27⋅6 (sd: 4⋅0)	0⋅041
Income			0⋅003
Low	18 (32⋅1 %)	22 (64⋅7 %)	
High	38 (67⋅9 %)	12 (35⋅3 %)	
Children			0⋅255
Yes	52 (92⋅9 %)	29 (85⋅3 %)	
No	4 (7⋅1 %)	5 (14⋅7 %)	
Grandchildren			0⋅980
Yes	46 (82⋅1 %)	28 (82⋅4 %)	
No	10 (17⋅9 %)	6 (17⋅6 %)	
Educational level^b^			0⋅228
Low	6 (10⋅7 %)	4 (11⋅8 %)	
Middle	36 (64⋅3 %)	25 (73⋅5 %)	
High	14 (25⋅0 %)	3 (8⋅8 %)	

a Older adults with a score >30 % are considered to be at high risk for low protein intake (<1⋅0 g/kg bw/d).

b Educational level was defined as ‘Low’ when participants had only completed primary education, lower vocational education and/or advanced elementary education; ‘Middle’ for completing intermediate vocational education, higher secondary education; and ‘High’ for higher vocational education and university.

### Focus groups (study 1)

The outcomes of the focus groups are divided into two main topics: healthy eating and attitude towards high protein products. All results shown are a reflection of answers as given by the participants, and summarised by the researchers.

#### Healthy eating

Participants were asked for their current grocery shopping pattern, and to what extent their current stage of life had an influence on food habits, in order to gain more insights into the role of the aspect of health on their daily behaviour. All participants indicated that they did their own groceries in the supermarket, sometimes supported by family or friends. They preferably visited the nearest supermarket, due to physical limitations. The term ‘healthy eating’ was interpreted as frequent consumption of fruits and vegetables. Some respondents mentioned protein-rich products, because they had read that it was healthy for them. The participants did not know exactly why these products are claimed to be healthy for them. Although many respondents indicated they used some kind of supplements (e.g. vitamins, calcium), none of them used protein supplements (such as protein powders or high protein shakes). Half of the participants indicated that they had adapted their eating behaviour, due to the ageing process. Participants with a partner more often set the table when having dinner, while single participants more often ate while watching television. Nutritional adaptations that participants had made during the course of their life that were frequently mentioned were: less salt, less fat and increased protein intake, although for some participants using less salt in relation to healthy eating is more common, and not necessarily related to ageing [‘*I used my saltshaker for over 35 years*’]. Using fewer ready-to-eat sauces or herbs mixes and avoiding ready-to-eat hot meals were methods mentioned in order to decrease salt intake [‘*I don't use ready to eat meals or herbs mixes, to avoid water retention*’]. Some older adults stated that, due to high cholesterol levels, they had decreased their fat consumption, by replacing regular products with skimmed products and decreased consumption of pork. Although some participants rejected an increased protein consumption [‘*I turned 91 years of age with my usual dietary pattern, so why should I bother about protein?*’], others said to have increased the consumption of products high in protein, such as yoghurt, milk, eggs, fish and chicken. The main reason for this change was upon recommendation by a medical specialist, dietitian or because they had read that protein supports muscle synthesis [Subject 1: ‘*Protein is good for building muscle*’. Subject 2: ‘*I used to be very active, it is good for that too*’.]. Medical specialists and dietitians were also indicated to be the most reliable source for information on healthy eating [‘*I started to eat more protein as advised by my dietitian*’; ‘*You can have a yearly check-up with the GP and then they check everything, including vitamin blood levels*’.]. Some participants also mentioned other sources of information, such as family and friends or food-related television programmes, and some indicated to do an independent internet search on healthy eating.

#### Attitude towards high protein products

To assess the attitude of the participants towards high protein products, participants were presented a range of commercially available products with different health claims. Discussions revealed that the main driver to consume foods/products is palatability. Only margarine containing plant sterols was specifically consumed because of the claimed health benefits. However, older adults indicated that a health claim is not a reason to buy, since they believed that they already had sufficient knowledge about nutrition to know what they need [‘*one time yes, other times no. Sometimes I also think it's solely for the money. Sometimes I just do what I want. It keeps changing what is good and what is not. I just have to listen to myself carefully*’.]. Some participants even indicated to mistrust information on products, and preferred verification with a medical specialist or on the internet. Participants mentioned that this distrust was caused by various food safety scandals that had happened in the past and was supported by different television programmes [‘*I don't rely on health claims on products. I want to be sure and check for myself. Product labels are often incorrect*’]. When comparing the offered products with the health claims, the products high in protein were considered to be most healthy by the participants, since, as described previously, an increase in protein intake was thought to be important. The use of athlete photos on high protein product packages did not support this acknowledged importance: it was even suggested to serve adversely as reliability of these products was questioned [Subject 1: '*Those athletes just earn money with that.'* Subject 2: '*And then it is not yet certain that he uses it.*’]. Finally, none of the participants indicated the need for enriched products, such as milk with added calcium, fibres in muesli, yoghurt products with added protein, since they considered themselves sufficiently skilled to compose a healthy diet containing all nutrients. Therefore, enrichment of food products was not seen as requirement.

### Photovoice (study 2)

Based on the pictures of thirty subjects, it became clear that almost all participants consumed their meals at the dinner table and that they set the table before having a meal. Upon asking, most participants indicated that they value the meal moments and they take time to have a meal. Analysis of time stamps on the pictures showed that most participants followed a regular pattern regarding timing of meal consumption, although in the weekend, breakfast was consumed a bit later.

A traditional Dutch dietary pattern (as defined in^(34)^) was identified among sixteen out of thirty participants, meaning that these older adults preferably consumed traditional products such as potatoes, dairy, fruits and cheese or meat on their sandwiches and 98 % of the participants indicated not to be willing to change their current eating behaviour (‘*I am very satisfied as it is*’). The group of participants with a less traditional eating pattern appeared to have more variation in warm meals and consumed a larger variety in side dishes. Only one participant said that, due to taking pictures, on some photos behaviour was modified.

### Interviews on barriers and promotors of adequate protein intake (study 3)

Outcomes of the interviews regarding barriers and promotors for the use of high protein products were categorised into the themes ‘person’, ‘environment’ and ‘product’ and analyzed for single (*n* 10) and married (*n* 10) older adults separately.

Although no data saturation was reached, these results provide additional insights on possible barriers and promotors of protein intake in older adults. A summary of findings is presented in Table 4.

**Table 4.** Main barriers and promotors for the use of high protein products as identified in interviews with both single and married older adults. Items mentioned were identified as either barrier or promotor in both groups

Main barriers	Main promotors
*Person*	*Person*
Physical and mental deterioration	Variation in dietary pattern
Lack of knowledge	Trust in professionals
Resistance to change habits	No difficulties with food preparation and consumption
*Environment*	*Environment*
Difficulties in supermarket	Social interaction in supermarket
Changing information on healthy diet	Open for dietary counselling when necessary
No dietary advice on protein	
*Product*	*Product*
Product packaging	Product offers

#### Person-related barriers and promotors

An example of a mentioned barrier at a personal level was physical and mental deterioration, causing lower physical activity and decreased mobility [‘*My car is very important to me. That's my freedom*’]. Physical deterioration could also lead to dependency on walking aids, subsequently causing difficulties since some participants indicated that they had to limit buying the amount of products when doing grocery shoppings [‘*I can't bring along as much as I used to. My walker fills up faster than a shopping cart.*’]. This results in more frequent shopping occasions, for which additional barriers and promotors were identified (see Section ‘Environment’). Another major barrier for the use of high protein products is the lack of knowledge, incorrect knowledge and lack of interest in the topic. Some participants indicated that they do not feel the urge to change their behaviour at their age [‘*I don't worry about that anymore*’]. Some participants declared to have no knowledge on protein at all, and other participants thought to have some knowledge, but we interpreted this knowledge as being wrong or incomplete [‘*14 days ago we were tested for protein, we had a finger prick and had to raise our arm*’]. From a ‘person’ perspective, the product variety could act as a promotor for married older adults, because when people are together they are more willing to try new products [‘*if we go shopping together, we're open to try something new*’].

In general, participants experienced no difficulties in the preparation and consumption of food products, although in some cases, single subjects did not feel the need to prepare extensive dinners. Furthermore, many participants indicated that leftovers are not spilled, but stored for the next day or put in the freezer.

#### Environment-related barriers and promotors

Distance to the nearest supermarket was considered to be either a barrier (large distance) or a promotor (supermarket is nearby). Supermarkets play an important role in the environment, since they can stimulate social interaction and inspire participants. On the contrary, the large amount of products [‘*Sometimes I offer to bring along a product for a friend, but when in the supermarket I see so many variations and then I'm lost*’], as well as the number of people present can act as a barrier. To avoid crowded places, some participants indicated to do their grocery shopping during specific timeslots.

Participants indicated that much information on a heathy dietary pattern is available in their environment, but it was considered to be a barrier that they observe changes in recommendations continuously [‘*Three years ago, eggs were considered to be bad, and now you can have 3 eggs a day without health consequences*’]. Furthermore, only a few participants indicated to receive dietary counselling, but they expressed their confidence in healthcare professionals.

#### Product-related barriers and promotors

From a ‘product’ perspective, participants often mentioned the aspect of packaging. Some older adults indicated that some products are difficult to open, but they also mentioned that, in most cases, they found a way to solve their problems. Portion size, as determined by the size/volume, was considered to be a promotor, for example, due to shelf life. For some participants, a small portion size was seen as a barrier, due to the given portion size [‘*I don't take these small packages, then you have to eat it all*’].

The majority indicated that product offers are definitely a promotor for the use of products and some of the older adults also mentioned that they are more willing to buy unfamiliar products when these are on sale.

### Knowledge and communication channels (study 4)

Forty older adults participated in interviews on knowledge of dietary protein and suitable communication methods. The aim was to identify optimal strategies to improve protein intake via knowledge, as one of the determinants of behaviour. Outcomes of these interviews confirmed the finding of study 3 that knowledge of dietary protein is relatively limited, since nineteen out of forty indicated to have no or only little knowledge of protein when asked ‘what do you know about protein?’ [‘*Not so much, I believe they are present in eggs and in vegetables*’ or ‘*Very little, I don't think about that. If I like something I buy it, otherwise I don't*’]. Other participants were able to mention at least some products high in protein (such as eggs, dairy, meat and nuts), and a few older adults responded with ‘*protein is good for your muscles and bones or for your blood*’ or that ‘*protein is present in food and you need them*’.

Prior to providing information on dietary protein requirements, participants were asked if they knew how much protein they need every day. All participants indicated that they were not aware of exact amounts per day, and twenty-three older adults made a guess with outcomes ranging from 2 to 300 g/d. After being informed on dietary requirements, twenty-four out of forty older adults stated that they think they consume sufficient amounts of protein. Whether participants actually met their dietary requirement was measured through the outcomes of the Pro55+^(30)^. If participants say ‘Yes’, they should score ‘low’ on the Pro55+, which was the case in 13/24 cases. On the other hand, if they say ‘No’, they should score ‘high’ (>30 %) on the Pro55+, which happened in 10/16 cases. In other words, seventeen out of forty had no good indication of their daily protein intake.

In order to define optimal communication strategies, older adults were asked for their preferred communication channels. All participants indicated to watch television frequently and most older adults (33/40) listened to the radio on a daily basis. Other communication tools included internet, smartphone and computer, with tablets as least popular used tools. Furthermore, thirty-five out of forty participants mentioned newspapers as a familiar communication tool, with a preference for regional newspapers instead of national daily newspapers.

## Discussion

The present study aimed to identify determinants, barriers and promotors with regard to an adequate protein intake among Dutch CDOA. In addition, we investigated opportunities for modification of their current dietary behaviour in order to increase protein intake. The results of the present study show that almost one third of the participants had a high risk of a too low protein intake (<1⋅0 g/kg bw/d), which is more common with low income. Participants often stated that mainly fruits and vegetables are important for a healthy diet, but they were mostly unaware of the importance of dietary protein, or products that contain protein. Accordingly, older adults were often unaware of the increased protein requirements during an older age and in general, they tended to overestimate their daily protein intake. However, they usually indicated to pay attention to healthy eating and consider meal moments as very important; meal atmosphere is valuable to them and they tended to have a regular meal pattern. A traditional Dutch diet containing hardly any novel foods was common and many participants were not willing to change their diet drastically in order to improve their health. However, there are also less traditional older adults who are more open to new food products. Some important barriers with regard to consuming an adequate amount of dietary protein are physical and mental deterioration and lacking knowledge. Promotors that were identified are product offers and that older adults express their trust in advice from healthcare professionals. To increase awareness about the importance of protein intake, suitable communication channels might be television and radio, since most older adults watch and listen to these channels on a daily basis. With regard to newspapers, they prefer regional to national newspapers. Medical specialists and dietitians are regarded as most reliable sources of information, while photos of athletes on foods might decrease perceived reliability of foods.

Participants from the present study are considered to be a proper reflection of the Dutch CDOA population, so outcomes of the present study may be attribute to the overall aim of reducing malnutrition among CDOA. Participants from the ConsuMEER study (study 1) were considered to be comparable with other large Dutch cohorts, such as the Dutch Longitudinal Ageing Study Amsterdam (LASA) cohort^(35)^ and the Dutch National Food Consumption Survey Older Adults (DNFCSOA)^(17)^. The participants in studies 2, 3 and 4 were slightly younger than the ConsuMEER participants with a mean age of 75⋅6 y ± 7⋅8 compared with 80⋅4 y ± 6⋅8^(29)^. The average BMI of ConsuMEER (study 1) participants was 27⋅9 ± 5⋅0, which is a bit higher than the participants from studies 2, 3 and 4 (mean BMI 26⋅6 ± 3⋅5 kg/m^2^).

Participants from the focus group interviews indicated that enrichment of food products was not required, since they consider themselves sufficiently skilled and knowledgeable. This is in contrast with a previous study by Beelen *et al.*, which already described that older adults have insufficient knowledge about undernutrition and its health consequences^(28)^. Beelen *et al.* indicated that particularly enrichment of familiar foods was a promising strategy to increase protein intake^(36)^. Based on focus group outcomes, it can be discussed if older adults are indeed sufficiently skilled, or more important, they are apparently unaware of this knowledge gap. Focus group participants said that they had increased the consumption of high protein products, but indicated that was mainly upon recommendation by a medical specialist or dietitian. It is not known if participants in our studies received more dietary counselling than participants from other studies, as nutritional interventions have shown to be successful in the treatment of malnutrition and might attribute to this increased use of high protein products^(37)^. In the interviews, dietary counselling was also mentioned as a possible promotor for the use of high protein products. The aspect of knowledge was further explored in a set of semi-structured interviews (study 4) and, in these interviews, it was found that older adults have limited knowledge of dietary protein. The absence of knowledge of protein may be partially explained by the finding that, in studies 2, 3 and 4, more participants had either a low or middle educational level, comparable with previous research where also a greater proportion of participants was lower educated and knowledge of protein was low^(27)^. However, a lower educational level was not associated with a difference in the prevalence of low protein intake in another study^(38)^. Since a lack of knowledge and a lack of interest on the topic were also identified as a barrier for the use of high protein products, future studies should incorporate the aspect of knowledge to determine how this aspect may affect dietary behaviour, and how knowledge could be increased to make older adults more aware of their risk on low protein intake. However, a limitation in the interviews on barriers and promotors is that no data saturation occurred, partially explained by the recruitment of subjects. Participants were recruited mainly in rural areas, but one participant was accidently recruited in the city, which led to new unique responses and thus no data saturation. Furthermore, a limitation may be that focus group interviews were held only with volunteers from the ConsuMEER study, and thus, data may not be completely saturated, outcomes provided ample insights in underlying causes which were further validated in studies 2, 3 and 4.

With regard to optimising protein intake, it is also important to know the current daily dietary patterns, and to identify opportunities at different meal moments. In general, it was shown that, in CDOA, the daily protein intake is in line with current dietary recommendations (>0⋅8 g/kg bw/d)^(16)^. More specifically, the current intake during the warm meal moment often adheres to dietary guidelines although interventions at this meal moment, with subjects switching from cooking to home-delivered hot meals, may introduce a risk for a low protein intake^(29)^. Such adverse outcomes could partially be explained by the absence of personal motivation to increase protein intake, as mentioned in both focus group interviews and additional interviews. In the present study, it also became clear that older adults are not aware of the possible health effects of a low protein intake and that many of them indicated that they were not willing to change their current dietary behaviour, which is in line with previous research^(39)^. Different models of behaviour have suggested that knowledge might be a large influence on behaviour (e.g. the I-Change model^(40)^). From the interview data, it becomes clear that awareness among older adults on low protein intake is limited and improvement of knowledge could support strategies to reduce the prevalence of malnutrition. In order to get recommendations across, older adults prefer communication via television, radio or local newspapers. Information should, however, comprise a consistent message on the benefits of dietary protein for older adults, to promote the consumption of higher protein products.

In studies 2, 3 and 4, the risk of low protein intake was assessed using the Pro55+, an online tool that was validated among comparable populations^(30)^. It appeared that more than a third of the participants had a high risk of low protein intake, with a cut-off of 1⋅0 g/kg bw/d. It could, therefore, be discussed how accurate older adults can describe their current behaviour, as it was also shown in the interviews that they have no good indication of their daily protein intake. This may suggest that older adults are insufficiently aware of necessary modifications to their current behaviour to adhere to the current dietary guidelines. To further elaborate on this aspect, photovoicing was applied which showed capabilities to provide additional insights into their real behaviour, such as showing that almost all participants consumed their meal at a set table. Furthermore, it was possible to identify participants with a traditional pattern, which has been shown before^(28)^, and it also provided more divergent insights into dietary behaviour such as timing of meals and eating behaviour.

Photovoicing, as applied in the present study, cannot be used to quantify intake volumes accurately. Limitations of this technique are that portion size could not be determined exactly, and that the photos might not show total consumption (e.g. in between snacks), causing an underestimation of protein intake based on solely photo data. Even though it might be possible that some consumption might not be photographed, the photos do show a complete overview of the three main meal moments (breakfast, lunch, warm meal), as these are the main contributors to daily protein intake^(16)^. The main reason to apply this new method in the present study was that previous studies have shown that participants may provide socially desired responses in interviews^(41)^. Noteworthy is that only one participant indicated in the interview that behaviour was modified on some photos, so the majority of the photos should reflect actual behaviour. Future studies could optimise the protocol to enhance the value of photos alongside common dietary intake methods.

It was suggested by Borkent *et al.* that qualitative research was needed to investigate how older adults could be motivated to increase the consumption of high protein products and how they could be supported in changing dietary habits^(29)^. Altogether, the outcomes of these four qualitative studies provide deeper insights into the attitudes of older adults towards high protein products and some main barriers and promotors for protein intake have been identified. Knowledge on protein, as determinant of behaviour, appeared to be rather limited. Taking into account that the typical older adult does not exist as identified in other studies^(42)^, more research is required to further investigate various behavioural aspects and how knowledge possibly could induce a change in dietary behaviour of older adults.

## Conclusion

It can be concluded that older adults have no urgency to increase protein intake, possibly linked to low or incorrect knowledge on the importance of protein and the overestimation of their own dietary protein intake. One of the challenges for professionals would be to increase knowledge and awareness, motivate older adults to change their eating pattern and optimise protein intake, in a personalised approach. Based on several barriers and limitations, supermarkets could play a key role in promoting the use of high protein products. However, to increase the chance of a successful change, solutions should fit with the current eating patterns.

## Supplementary material

For supplementary material accompanying this paper visit https://doi.org/10.1017/jns.2021.92. click here to view supplementary material
