# Supplementary material for: Low awareness of community-dwelling older adults on the importance of dietary protein: new insights from four qualitative studies
Source: J Nutr Sci. 2021 Dec 7;10:e102. doi: 10.1017/jns.2021.92 (PMC8727701; doi:10.1017/jns.2021.92)
Supplement: Supplementary file 1 [file S2048679021000926sup001.docx]

**Supplementary data**

Ad. 2.2: ProteinScreener55+

**PROTEIN SCREENER**

Wijnhoven HAH, Elstgeest LEM, de Vet HCW, Nicolaou M, Snijder MB, Visser M (2018) *Development and validation of a short food questionnaire to screen for low protein intake in community-dwelling older adults: The Protein Screener 55+ (Pro55+)*. PLoS ONE 13(5):e0196406

*The following questions are about your dietary habits. It is very important that you give an honest response. We would like to know what you ate or drank in the last 4 weeks (irrespective of week days, weekend days, at home or someplace else). If the last 4 weeks were very special (for example you were sick or you went on a vacation and this had a major influence on your usual diet), please recall the 4 weeks before this period.*

**Please enter your body measurements**

Age [year]

Weight [kilograms]

Length [centimeters]

*In the following questions, we ask how much of a food product you ate.*

**Question 1**

In the last 4 weeks, how many slices of bread did you eat on an average day?

- Less than one or not applicable
- 1 slice
- 2 slices
- 3 slices
- 4 slices
- 5 slices
- 6 slices
- 7 slices
- 8 slices
- 9 slices
- 10 slices
- 11 slices
- 12 slices
- > 12 slices

**Question 2**

In the last 4 weeks, how many glasses/cups of milk, buttermilk or soy milk did you drink on an average day?

- Less than one or not applicable
- 1 glass
- 2 glasses
- 3 glasses
- 4 glasses
- 5 glasses
- 6 glasses
- 7 glasses
- 8 glasses
- 9 glasses
- 10 glasses
- 11 glasses
- 12 glasses
- > 12 glasses

**Question 3**

How much meat did you on average eat on a day that you ate meat with your warm meal in the last 4 weeks?

- Not applicable, does not eat meat
- 1/5 plate
- 1/4 plate
- 1/2 plate
- 2/3 plate
- 3/4 plate

*In the following questions, we will ask how often you ate a certain product.*

**Question 4**

In the last 4 weeks how often did you yoghurt, quark, milk-based pudding, or soy dessert?

- Not in these 4 weeks
- 1 day in 4 weeks
- 2-3 days in 4 weeks
- 1 day/week
- 2 days/week
- 3 days/week
- 4 days/week
- 5 days/week
- 6 days/week
- 7 days/week

**Question 5**

In the last 4 weeks how often did you eat egg with either your breakfast, lunch, evening meal, as a snack, or in a meal?

- Not in these 4 weeks
- 1 day in 4 weeks
- 2-3 days in 4 weeks
- 1 day/week
- 2 days/week
- 3 days/week
- 4 days/week
- 5 days/week
- 6 days/week
- 7 days/week

**Question 6**

In the last 4 weeks how often did you eat pasta or noodles (like spaghetti, macaroni, lasagna, chow mein, rice-based or wheat-based noodles)?

- Not in these 4 weeks
- 1 day in 4 weeks
- 2-3 days in 4 weeks
- 1 day/week
- 2 days/week
- 3 days/week
- 4 days/week
- 5 days/week
- 6 days/week
- 7 days/week

**Question 7**

In the last 4 weeks how often did you eat fish with your bread meal, warm meal, or as a snack? (Do NOT include shellfish).

- Not in these 4 weeks
- 1 day in 4 weeks
- 2-3 days in 4 weeks
- 1 day/week
- 2 days/week
- 3 days/week
- 4 days/week
- 5 days/week
- 6 days/week
- 7 days/week

**Question 8**

In the last 4 weeks, how often did you eat nuts or peanuts as a snack?

- Not in these 4 weeks
- 1 day in 4 weeks
- 2-3 days in 4 weeks
- 1 day/week
- 2 days/week
- 3 days/week
- 4 days/week
- 5 days/week
- 6 days/week
- 7 days/week

**Question 9**

In the last 4 weeks how often did you eat cheese or cheese spread on your bread, bun, rusk, cracker, etc.?

- Not in these 4 weeks
- 1 day in 4 weeks
- 2-3 days in 4 weeks
- 1 day/week
- 2 days/week
- 3 days/week
- 4 days/week
- 5 days/week
- 6 days/week
- 7 days/week

**Question 10**

How many slices of bread, bun, rusk, cracker, etc. with cheese or cheese spread did you on average eat on a day that you ate cheese or cheese spread?

- Less than one or not applicable
- 1 slice
- 2 slices
- 3 slices
- 4 slices
- 5 slices
- 6 slices
- 7 slices
- 8 slices
- 9 slices
- 10 slices
- 11 slices
- 12 slices
- > 12 slices

*Predicted Probability:*

*XX%*

*We consider a chance of more than 30% as a high risk on a low protein intake.*

*To more precisely measure actual protein intake, a full nutritional assessment should be done by for example a dietitian.*

Ad. 2.3.2: Photovoice interviews

| Post-photovoice interview questions |
| --- |
| - Are you satisfied with your current dietary pattern with regard to health? - Would you like to modify your current dietary pattern with regard to health? - Where do you usually consume your warm meal? - Where do you usually consume your other meals? - Do you usually consume your warm meal together with others? - Do you usually consume your other meals together with others? - Are there things you would like to change when consuming your meals? - How many days per week do you eat out of home? - How many days per week would you like to eat out of home? - How many days per week do you prepare your own meal? - How many days per week would you like to prepare your own meals? - Are there any foods that you consciously avoid? - To what extent are you aware that eating enough protein is important for your health? |
